# The effect of neoadjuvant chemotherapy on ductal carcinoma in situ in triple-negative breast cancer patients: A nationwide analysis

**DOI:** 10.1016/j.breast.2025.104425

**Published:** 2025-02-18

**Authors:** Eva L. Claassens, Roxanne A.W. Ploumen, Loes F.S. Kooreman, Maartje A.C.E. van Kats, Sabine Siesling, Thiemo J.A. van Nijnatten, Marjolein L. Smidt

**Affiliations:** a Department of Surgery, Maastricht University Medical Centre+, Maastricht, the Netherlands; b GROW – Research Institute for Oncology and Reproduction, Maastricht University, Maastricht, the Netherlands; c Department of Pathology, Maastricht University Medical Centre+, Maastricht, the Netherlands; d Department of Medical Oncology, Maastricht University Medical Centre+, Maastricht, the Netherlands; e Department of Radiology and Nuclear Medicine, Maastricht University Medical Centre+, Maastricht, the Netherlands; f Department of Health Technology and Services Research, Technical Medical Centre, University of Twente, Enschede, the Netherlands; g Department of Research and Development, Netherlands Comprehensive Cancer Organization (IKNL), Utrecht, the Netherlands

**Keywords:** Ductal carcinoma in situ, Neoadjuvant chemotherapy, Pathologic complete response, Triple-negative breast cancer

## Abstract

**Purpose:**

Recent studies show that ductal carcinoma in situ (DCIS) accompanying HER2+ breast cancer can be completely eradicated following neoadjuvant systemic therapy in up to 52 %. We aimed to determine the complete response rate of DCIS in triple-negative breast cancer (TNBC) patients in a nationwide cohort and to assess clinicopathological variables associated with response. Furthermore, the impact on surgical treatment after neoadjuvant chemotherapy (NACT) was investigated.

**Methods:**

Women diagnosed with TNBC, treated with NACT followed by surgery, between 2010 and 2020, were selected from the Netherlands Cancer Registry (NCR). Pre-NACT and postoperative pathology reports were obtained from Palga, the Dutch nationwide pathology databank, to determine presence of DCIS. Clinicopathological factors associated with DCIS response were investigated using uni- and multivariable logistic regression analysis.

**Results:**

In total, 4494 patients were included. A DCIS component was present in the pre-NACT biopsy of 442 (9.8 %) patients. Pathologic complete response of the DCIS component was achieved in 53.6 % of these patients. The presence of calcifications in the pre-NACT biopsy was associated with a lower chance of DCIS response in univariable logistic regression analysis (OR 0.52, CI 95 % 0.27–0.98, p = 0.04). In multivariable analysis, no statistically significant associations were found between DCIS response and clinicopathological variables. Mastectomy rates were higher in case of IBC + DCIS compared to IBC (53.4 % vs 40.1 %, p < 0.001).

**Conclusion:**

Pathologic complete response of DCIS to NACT occurred in 53.6 % of TNBC patients. Future studies are required to be able to predict DCIS response based on clinicopathological variables and imaging.

## Highlights

• Ductal carcinoma in situ present in 9.8 % of triple-negative breast cancer biopsies.
• Nationwide analysis of ductal carcinoma in situ response to neoadjuvant chemo.
• Pathologic complete response of ductal carcinoma in situ occurred in 53.6 %.
• Absence of calcifications in diagnostic biopsy associated with complete response.
• Mastectomy rates significantly higher in case of adjacent ductal carcinoma in situ.

## 1 Introduction

The role of neo-adjuvant systemic therapy (NST) in the treatment of invasive breast cancer (IBC) has evolved significantly. NST allows for in vivo evaluation of tumour response and has the potential to downstage the extent of the disease in the breast and/or axilla, thereby improving oncologic outcomes as well as enabling breast-conserving surgery [1]. Previously, ductal carcinoma in situ (DCIS) was considered to be unresponsive to systemic treatment [2]. Therefore, the presence of DCIS in IBC patients poses a challenge to the potential of de-escalating breast surgery after NST.

Multiple studies have focused on the response of DCIS to NST in HER2-positive IBC, mainly since a DCIS component is often present in HER2-positive IBC patients [3]. A recent nationwide analysis of 1403 patients found a pathologic complete response (pCR) of 52.0 % for DCIS in HER2-positive IBC patients following NST [4]. Despite DCIS occurring less frequently in triple-negative breast cancer (TNBC) patients (34.1–47.2 %) than in HER2-positive patients (57.4–71.6 %), both HER2-positive and triple-negative (TN) IBC tend to respond well to systemic therapy [3,[5], [6], [7]]. Approximately 43–55 % of patients with TNBC achieve pCR after neoadjuvant chemotherapy (NACT), compared to 46–68 % in HER2-positive patients [[8], [9], [10], [11], [12]]. Considering that TNBC generally responds favorably to NACT, it is possible that the accompanying DCIS component may show a similar response to NACT. Van la Parra et al. investigated this possibility and demonstrated that in their study population of 78 TNBC patients with a DCIS component, 45 % of DCIS was eradicated following NACT [13].

Nevertheless, data on the response of DCIS to NACT in TNBC patients remain limited as it has only been investigated in a small study cohort. Therefore, the aim of this study was to determine pCR rate of the DCIS component accompanying TNBC in a nationwide cohort. Furthermore, the association between pCR of TN IBC and pCR of the accompanying DCIS component was investigated as well as the potential association between clinicopathological variables with pCR of DCIS. Additionally, the impact of the DCIS component on surgical treatment was assessed.

## 2 Methods

### 2.1 Data sources and study population

The Netherlands Comprehensive Cancer Organization (IKNL) assembles data regarding patient, tumour and treatment characteristics of all newly diagnosed cancer patients to provide the nationwide Netherlands Cancer Registry (NCR). After request for data and approval of the Committee of Privacy of the NCR (application number K24-00482), the assembled data can be used for research, as performed in this study. Women aged 18 years or older, diagnosed with TNBC, treated with NACT followed by surgery between January 2010 and December 2019 in the Netherlands were selected from the NCR. From Palga, the Dutch nationwide pathology databank, all pre-NACT biopsy and postoperative specimen pathology reports of the selected patients from the NCR were obtained [14]. Data from the NCR and Palga were combined to form the database used for the current study. Patients were excluded in case of missing biopsy or postoperative pathology reports and when patients diagnosed with a cTIS or cTX received NACT despite being cN0 (i.e. without cN + being the reason to receive NACT).

### 2.2 Neoadjuvant chemotherapy and surgical procedure

NACT regimens were based on the Dutch NABON Breast Cancer Guidelines. In TNBC patients, NACT is recommended in case of tumour size ≥ 1 cm and/or biopsy-proven node-positivity [15]. In general, the NACT regimen consisted of Anthracyclines and Taxanes. From 2014 onwards, treatment typically consisted of (dose dense) AC followed by Paclitaxel, with or without Carboplatin depending on the treating center. Surgical options regarding breast surgery following NACT included breast-conserving surgery (BCS) and mastectomy, with the final decision made by the treating surgeon in consultation with the patient.

### 2.3 Assessment of pathologic response

From the NCR, data including IBC morphology, grade and receptor status were obtained. IBC grade was assigned according to The Bloom-Richardson Grading System [16]. Receptor status was determined for the IBC component, not for the DCIS component. HER2 status was determined by immunohistochemistry, in situ hybridization, or a combination of both, following the ASCO CAP guidelines [17]. The ER status was assessed using immunohistochemistry and considered positive if ≥ 10 % of the tumour cells showed positive staining. Pathological examination was conducted locally in accordance with Dutch guidelines [15]. Resection specimens weighing less than 30 g were fully embedded for microscopic examination. For larger specimens, at least one slide per centimeter of the anticipated tumour region was prepared for analysis. Most laboratories use the Dutch Pathology Database (Palga) protocol for synoptic reporting to facilitate clear and concise reporting. The presence of a DCIS component was assessed in the pre-NACT and postoperative pathology reports, received through Palga. Additionally, the grade of the DCIS component and the presence of comedonecrosis and/or calcifications were evaluated in the pre-NACT biopsy. When information regarding the grading, presence of comedonecrosis and/or calcifications was not reported on, this was recorded as ‘missing data’. Adjacent DCIS was defined as presence of DCIS in the pre-NACT biopsy and/or in the postoperative specimen. Our analyses includes DCIS diagnosed both within the same biopsy as the invasive tumour and in any additional biopsy taken pre-NACT. Patients with DCIS in the pre-NACT biopsy were included in the DCIS response analysis. For both the DCIS and the invasive component, response was defined as pCR, meaning eradication of the DCIS or invasive component in the postoperative specimen.

### 2.4 Study objectives

The primary endpoint was the percentage of pCR of DCIS in TNBC patients with a DCIS component in the pre-NACT biopsy. Secondary endpoints included the correlation between clinicopathologic variables and pCR of DCIS, the association between pCR of IBC and pCR of DCIS and the impact of the presence of a DCIS component on surgical treatment type.

### 2.5 Statistical analysis

Missing data for the variables IBC grade and DCIS grade were considered to be missing at random and were therefore imputed using multiple imputation, forming ten imputed datasets. Rubin's rules were subsequently applied to combine the results from the ten imputed datasets. [18] Patient characteristics were analyzed with descriptive statistics. Continuous variables are represented as median and range, categorical variables are represented as counts and percentages (%). Pearson's chi-square test was used to compare characteristics of categorical variables. The influence of clinicopathological variables on the response of DCIS was evaluated by uni- and multivariable logistic regression analyses. A p-value ≤0.05 was considered statistically significant. Statistical analyses were performed using SPSS Version 29.

## 3 Results

### 3.1 Study population

**Fig. 1:**
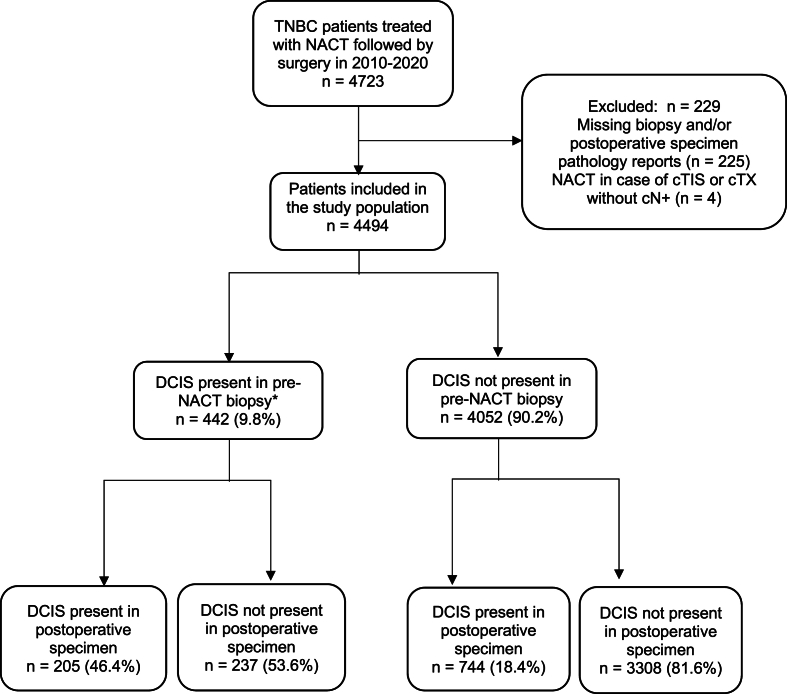
Flowchart of patient selection. ∗included in DCIS response analysis.

In total, 4723 women with TNBC were treated with NACT followed by surgery between 2010 and 2019 in the Netherlands. After exclusion of 229 ineligible patients, 4494 patients were included in the study population (Fig. 1). DCIS was present in the pre-NACT biopsy of 442 (9.8 %) patients. Subsequently, these patients were included in the DCIS response analysis.

### 3.2 Patient characteristics

Of the 442 patients with IBC with an adjacent DCIS component, the majority was diagnosed with a cT2 (57.9 %) tumour and histopathology of no special type (90.0 %) (Table 1). The invasive tumour as well as the DCIS component was most commonly grade 3, in 62.7 % and 70.8 % respectively. Comedonecrosis was found in 68.1 % and calcifications associated with the DCIS component was found in 47.7 % of the pre-NACT biopsies.

**Table 1:** Baseline characteristics of patients with adjacent DCIS.

Characteristics	DCIS *n (%)* n = 442
Age at diagnosis in years, median [range]	48 [24–78]
Year of diagnosis	
2010–2013	91 (20.6)
2014–2016	141 (31.9)
2017–2019	210 (47.5)
Clinical tumour status	
T1	82 (18.6)
T2	256 (57.9)
T3	80 (18.1)
T4	21 (4.8)
Tis^a^	1 (0.2)
TX^a^	2 (0.5)
Clinical nodal status	
N0	233 (52.7)
N1	146 (33.0)
N2	14 (3.2)
N3	46 (10.4)
NX	3 (0.7)
IBC subtype	
No special type^b^	398 (90.0)
Lobular	2 (0.5)
Other	42 (9.5)
Grade IBC	
1	35 (7.9)
2	130 (29.4)
3	277 (62.7)
Grade DCIS	
1	19 (4.3)
2	110 (24.9)
3	313 (70.8)
Comedonecrosis	
Present	96 (68.1)
Absent	45 (31.9)
Missing	301
Calcifications	
Present	74 (47.7)
Absent	81 (52.3)
Missing	287

*DCIS* ductal carcinoma in situ*, IBC* invasive breast cancer.

a Diagnosed with cN+ and therefore treated with NACT.

b Formerly known as invasive ductal carcinoma.

### 3.3 Association between clinicopathologic variables and complete response of DCIS to NACT

As presented in Figs. 1 and 53.6 % of patients with DCIS in the pre-NACT biopsy showed a complete response of the DCIS component to NACT. Table 2 shows an overview of uni- and multivariable logistic regression analyses for clinicopathologic variables associated with pCR of DCIS. In univariable logistic regression analysis, presence of calcifications was significantly associated with a lower chance of the DCIS component achieving pCR (OR 0.52, CI 95 % 0.27–0.98, p = 0.04). Comedonecrosis and calcifications were not included in the multivariable logistic regression analysis given the high proportion of missing data. No statistically significant associations were found between the remaining clinicopathologic variables and DCIS response.

**Table 2:** Association of clinicopathological variables and IBC factors with complete response of DCIS to NACT in univariable and multivariable regression analyses.

		Univariable Analysis		Multivariable Analysis	
Clinicopathological variables	**Complete pathologic response of DCIS N/total (%)**	**OR (95 % CI)**	***p* value**	**OR (95 % CI)**	***p* value**
Age at diagnosis in years					
<50	133/241 (55.2)	REF		REF	
≥50	103/198 (52.0)	0.88 (0.60–1.28)	0.51	0.98 (0.66–1.45)	0.90
Year of diagnosis					
2010–2013	55/91 (60.4)	REF		REF	
2014–2016	69/141 (48.9)	0.63 (0.37–1.07)	0.09	0.66 (0.38–1.14)	0.13
2017–2019	112/207 (54.1)	0.77 (0.47–1.27)	0.31	0.75 (0.44–1.26)	0.28
Clinical tumour status					
T1	41/82 (50)	REF		REF	
T2	149/256 (58.2)	1.39 (0.85–2.29)	0.19	1.38 (0.83–2.30)	0.22
T3	36/80 (45.0)	0.82 (0.44–1.52)	0.52	0.84 (0.44–1.58)	0.58
T4	10/21 (47.6)	0.91 (0.35–2.37)	0.85	0.92 (0.33–2.53)	0.87
DCIS grade					
Grade 1 + 2	74/129 (57.4)	REF		REF	
Grade 3	162/310 (52.3)	0.81 (0.48–1.36)	0.42	0.67 (0.38–1.19)	0.17
IBC grade					
Grade 1 + 2	80/164 (48.8)	REF		REF	
Grade 3	156/275 (56.7)	1.37 (0.89–2.11)	0.15	1.55 (0.96–2.49)	0.07
Comedonecrosis					
Not present	21/44 (47.7)	REF			
Present	48/95 (50.5)	1.12 (0.55–2.29)	0.76		
Calcifications					
Not present	46/80 (57.5)	REF			
Present	30/73 (41.1)	0.52 (0.27–0.98)	0.04∗		

*OR* odds ratio, *REF* reference, *DCIS* ductal carcinoma in situ*, IBC* invasive breast cancer.

∗ = statistically significant.

^a^ Diagnosed with cN+ and therefore treated with NACT.

Table 3 shows the pathological outcomes of IBC and DCIS after NACT. A pCR of the DCIS component was achieved significantly more often in case of a pCR of the invasive component compared to residual IBC (70.6 % vs 40.0 %, p < 0.001).

**Table 3:** Pathological findings of IBC and DCIS after NACT.

N (%) Total n = 442	DCIS pCR n = 237/442 (53.6)	Residual DCIS n = 205/442 (46.4)	*p* value
			<0.001^a^

IBC pCR n = 197/442 (44.6)	139/197 (70.6)	58/197 (29.4)	

Residual IBC n = 245/442 (55.4)	98/245 (40.0)	147/245 (60.0)	

a statistically significant.

### 3.4 Surgical treatment

Mastectomy rates were significantly higher in case of IBC with an adjacent DCIS component in the pre-NST biopsy compared to IBC without DCIS (53.4 % vs 40.1 %, p < 0.001, Table 4). The postoperative pathology outcomes (ypT status) for IBC patients and IBC with adjacent DCIS patients, categorized by surgical treatment (BCS versus mastectomy), are shown in Table 5. In total, 1861 patients (41.4 %) were treated with mastectomy. Of these patients, 35.8 % achieved pCR (ypT0).

**Table 4:** Surgical treatment after NACT.

N (%) Total n = 4494	DCIS in pre-NACT biopsy n = 442	No DCIS in pre-NACT biopsy n = 4052	*p* value
			<0.001^a^

Breast-conserving surgery n = 2633 (58.6)	206/442 (46.6)	2427/4052 (59.9)	

Mastectomy n = 1861 (41.4)	236/442 (53.4)	1625/4052 (40.1)	

a statistically significant.

**Table 5:** ypT status per surgical treatment.

ypT status	Surgical treatment IBC		Surgical treatment IBC + DCIS		Total (n (%))
	Breast conserving surgery (n (%))	Mastectomy (n (%))	Breast conserving surgery (n (%))	Mastectomy (n (%))	
ypT0	1134 (46.7)	603 (37.1)	75 (36.4)	64 (27.1)	1876 (41.7)
ypTis	133 (5.5)	69 (4.2)	25 (12.1)	33 (14.0)	260 (5.8)
ypT1-2	830 (34.2)	535 (32.9)	44 (21.4)	42 (17.8)	1451 (32.3)
ypT1-2 + DCIS	306 (12.6)	197 (12.1)	61 (29.6)	74 (31.4)	638 (14.2)
ypT3-4	17 (0.7)	178 (11.0)	1 (0.5)	10 (4.2)	206 (4.6)
ypT3-4 + DCIS	6 (0.2)	37 (2.3)	0 (0.0)	12 (5.1)	55 (1.2)
ypTX	1 (0.0)	6 (0.4)	0 (0.0)	1 (0.4)	8 (0.2)
Total	2427	1625	206	236	4494

## 4 Discussion

In our study, an adjacent DCIS component to TNBC was found in the pre-NACT biopsy of 442 (9.8 %) of the included patients. We demonstrated that DCIS was completely eradicated following NACT in 53.6 % of these patients. In addition, in univariable analysis we found the presence of calcifications associated with DCIS in the pre-NACT biopsy to be associated with a lower chance of the DCIS component achieving pCR. Moreover, patients with a DCIS component in the pre-NACT biopsy were significantly more often treated with mastectomy compared to patients with IBC without DCIS.

In total, 4494 women with TNBC were analyzed of whom 442 (9.8 %) had an adjacent DCIS component in the pre-NACT biopsy. The incidence of DCIS in pre-NACT biopsies in TNBC patients is rarely reported in existing literature. However, our findings align with those of Labrosse et al., who observed a DCIS component in 9.5 % of pre-NACT biopsies of 359 TNBC patients [19]. In contrast, a higher incidence rate was found by Van la Parra et al. [13]. Their study investigated the effect of NACT on the DCIS component of TNBC patients. Of 328 patients in the population, 78 (23.8 %) patients had a DCIS component in the pre-NACT biopsy. The lower incidence of DCIS observed in pre-NACT biopsies compared to the reported overall DCIS rates in TNBC patients (34.1–47.2 %) may be explained by sampling limitations. Since DCIS can be located outside the invasive tumour, biopsies that primarily target the invasive component may miss the DCIS component, leading to underdetection. To the best of our knowledge, our study is the first nationwide analysis investigating DCIS response to NACT in TNBC patients. The pCR rate of 53.6 % that was demonstrated aligns with the outcome of the study by Van la Parra et al., who found a pCR of DCIS in 35/78 (44.9 %) patients [13]. Our study population is considerably larger, therefore the consistency in pCR rates may be seen as validation of the previous findings.

Data regarding DCIS response to NACT in TNBC patients is relevant as NACT frequently results in pCR of the invasive component, however the presence of extensive DCIS pre-NACT can reduce the possibility of BCS even when the invasive component achieved pCR. Our findings demonstrate that pCR of the DCIS component occurred significantly more often in case of pCR of the invasive component compared to residual IBC (70,6 % vs. 40.0 %, p < 0.001), suggesting that the response of the two components is related. This highlights the importance of identifying patients who are likely to achieve pCR of the DCIS component, as pCR of both the invasive and DCIS component is particularly beneficial for BCS. Therefore, we aimed to identify factors associated with DCIS response. This study is the first to explore the potential association between clinicopathological variables and DCIS response to NACT in TNBC patients. In contrast, several studies have already investigated similar associations in HER2+ breast cancer patients. Notably, both Groen et al. [20] and Ploumen et al. [4] found the absence of calcifications to be significantly associated with complete DCIS response, which aligns with the findings of the present study. This association was significant in multivariable analysis for Groen et al., while for Ploumen et al., absence of calcifications could not be included in multivariable analysis due to substantial missing data. In our univariable analysis, we found the absence of calcifications in pre-NACT biopsy to be associated with higher odds of the DCIS component achieving pCR (OR 0.52, CI 95 % 0.27–0.98, p = 0.04). However, presence of calcifications and/or comedonecrosis could not be included in multivariable analysis because of high numbers of missing data within these variables, resulting in too many patients being excluded from the analysis. The remaining investigated clinicopathological variables were not significantly associated with DCIS response in uni- and multivariable logistic regression analysis, possibly due to a sample size too small to achieve statistically significant results. Future studies with larger sample sizes are required to further investigate the association between clinicopathological variables and DCIS response.

Recently, studies have focused on the complete response of DCIS accompanying HER2-positive IBC and have demonstrated a pCR of DCIS in 36 %–52 % [4,20,21]. Nonetheless, previous research shows that patients with IBC accompanied by a DCIS component are more likely to undergo mastectomy than breast-conserving surgery, compared to patients with IBC without DCIS [4,7,22]. In our study population, mastectomy rates were significantly higher in case of IBC + DCIS compared to IBC without DCIS (53.4 % vs 40.1 %, p < 0.001, Table 4). However, this could not be further explored due to the lack of relevant data, such as extent of mammographic calcifications and patient preferences regarding surgical treatment.

Multiple studies have demonstrated that women treated with BCS generally report a higher quality of life and improved sexual well-being compared to women treated with mastectomy [[23], [24], [25]]. This finding should be considered in surgical decision-making and to support this process, research on evaluation of DCIS response on imaging is essential. A recent systematic review aimed to examine current literature on imaging findings evaluating DCIS response to NST. However, their findings state that calcifications on mammography can remain despite pCR of DCIS, and residual DCIS does not always show enhancement on MRI and contrast-enhanced mammography (CEM) [26]. This illustrates the current lack of reliable imaging characteristics to define DCIS response to NST. Nevertheless, in the case of non-mass enhancement on MRI/CEM, this can be considered a finding suspicious for malignancy, particularly DCIS [27,28].

This study has both strengths and limitations. First, the use of the nationwide NCR combined with the Dutch nationwide pathology databank allowed for large-scale evaluation of DCIS response. Secondly, various clinicopathological variables were investigated and the potential association of these variables with DCIS response was assessed. To our knowledge, we are the first to investigate the association between clinicopathological variables and DCIS response in TNBC patients. However, there are certain limitations worth mentioning. First, as the study involves a database of retrospective nature, slightly outdated chemotherapy regimens may have been used during the initial years of inclusion compared to the current standard. From 2014 onward, the regimen that is currently being used was implemented. Nonetheless, the data already demonstrate a substantial response rate of DCIS, which could possibly be even greater due to continual advancements in systemic therapy regimens [29]. Additionally, the retrospective nature resulted in a number of missing variables due to incomplete reporting, particularly in relation to the pathologic factors of the DCIS component. Multiple imputation was used to handle missing data within the variables IBC grade and DCIS grade. Despite the presence of a DCIS component in the postoperative specimen being a mandatory field in the Palga Protocol Module (PPM) since 2009, its presence in pre-NACT biopsies is not required for reporting. It has been included as an optional field since 2016, nevertheless this could have led to underreporting of the DCIS component. Second, the pre-NST biopsy collection forms another limitation. As mentioned earlier, DCIS can be located outside the invasive tumour, which presents the risk of the DCIS component being missed when the biopsy targets the invasive tumour. As a result, the presence of a DCIS component could be underestimated, which may have an impact on the observed response rate. Furthermore, the location of the DCIS component outside of the invasive component may affect the potential of performing BCS, however we could not evaluate this based on pre-NACT biopsy reports.

In conclusion, this nationwide retrospective study demonstrated a pCR of DCIS to NACT in 53.6 % of TNBC patients. Therefore, the presence of DCIS in TNBC patients should not necessarily diminish the eligibility for breast-conserving surgery. Future prospective studies are required to improve the prediction of DCIS response based on clinicopathological variables and imaging. The observed high mastectomy rates in patients with DCIS in our population emphasizes the need for further research to evaluate the possibility of de-escalating surgical interventions in patients who respond well to NACT.

## CRediT authorship contribution statement

**Eva L. Claassens:** Writing – review & editing, Writing – original draft, Visualization, Validation, Resources, Methodology, Investigation, Formal analysis, Data curation, Conceptualization. **Roxanne A.W. Ploumen:** Writing – review & editing, Resources, Methodology, Investigation, Data curation, Conceptualization. **Loes F.S. Kooreman:** Writing – review & editing, Methodology, Investigation, Data curation. **Maartje A.C.E. van Kats:** Writing – review & editing, Methodology, Investigation. **Sabine Siesling:** Writing – review & editing, Resources. **Thiemo J.A. van Nijnatten:** Writing – review & editing, Visualization, Resources, Methodology, Investigation, Conceptualization. **Marjolein L. Smidt:** Writing – review & editing, Visualization, Supervision, Resources, Methodology, Investigation, Conceptualization.

## Data availability statement

The data used for this retrospective study were obtained via The Netherlands Comprehensive Cancer Organization (IKNL) from the Netherlands Cancer Registry. This specific dataset is generated for the current study by the registration team of IKNL after permission by their Committee of Privacy (K24-00482). Therefore, the data are available upon reasonable request and with permission of the IKNL.

## Ethical approval

The current study was approved by the institutional ethics and privacy committee of The Netherlands Comprehensive Cancer Organization (IKNL) after reviewing the study protocol.

## Sources of funding

This research did not receive any specific grant from funding agencies in the public, commercial, or not-for-profit sectors. None of the authors received support from any organization for the submitted work.

## Declaration of competing interest

The authors declare the following financial interests/personal relationships which may be considered as potential competing interests: Marjolein L. Smidt reports a relationship with Nutricia that includes: funding grants. Marjolein L. Smidt reports a relationship with Servier Pharmaceuticals that includes: funding grants. Thiemo J.A. van Nijnatten reports a relationship with GE Healthcare that includes: funding grants and speaking and lecture fees. Thiemo J.A. van Nijnatten reports a relationship with Bayer that includes: funding grants and speaking and lecture fees. Loes F.S. Kooreman reports a relationship with SCEM that includes: speaking and lecture fees. Sabine Siesling reports a relationship with Pfizer that includes: funding grants. If there are other authors, they declare that they have no known competing financial interests or personal relationships that could have appeared to influence the work reported in this paper.
